# Comparison between Tail Suspension Swing Test and Standard Rotation Test in Revealing Early Motor Behavioral Changes and Neurodegeneration in 6-OHDA Hemiparkinsonian Rats

**DOI:** 10.3390/ijms21082874

**Published:** 2020-04-20

**Authors:** Ilaria Rosa, Davide Di Censo, Brigida Ranieri, Giuseppe Di Giovanni, Eugenio Scarnati, Marcello Alecci, Angelo Galante, Tiziana Marilena Florio

**Affiliations:** 1 Department of Life, Health and Environmental Sciences (MESVA), University of L’Aquila, 67100 L’Aquila, Italy; davide.dicenso@student.univaq.it (D.D.C.); ranieribrigida@gmail.com (B.R.); marcello.alecci@univaq.it (M.A.); angelo.galante@univaq.it (A.G.); tizianamarilena.florio@univaq.it (T.M.F.); 2 Laboratory of Neurophysiology, Department of Physiology and Biochemistry, Faculty of Medicine and Surgery, University of Malta, Msida MSD 2080, Malta; 3 Department of Biotechnological and Applied Clinical Sciences (DISCAB), University of L’Aquila, 67100 L’Aquila, Italy; eugenio.scarnati@univaq.it; 4 National Institute of Nuclear Physics, Gran Sasso National Laboratories, Assergi, 67100 L’Aquila, Italy; 5 SPIN-CNR Institute, Department of Physical and Chemical Sciences, 67100 L’Aquila, Italy

**Keywords:** Parkinson’s disease, 6-OHDA, animal models of Parkinson’s disease, apomorphine-induced behavior, tail suspension swing test, early motor behavior changes

## Abstract

The unilateral 6-hydroxydopamine (6-OHDA) model of Parkinson’s disease (PD) is one of the most commonly used in rodents. The anatomical, metabolic, and behavioral changes that occur after severe and stable 6-OHDA lesions have been extensively studied. Here, we investigated whether early motor behavioral deficits can be observed in the first week after the injection of 6-OHDA into the right substantia nigra pars compacta (SNc), and if they were indicative of the severity of the dopaminergic (DAergic) lesion in the SNc and the striatum at different time-points (day 1, 3, 5, 7, 14, 21). With this aim, we used our newly modified tail suspension swing test (TSST), the standard rotation test (RT), and immunohistochemical staining for tyrosine hydroxylase (TH). The TSST, but not the standard RT, revealed a spontaneous motor bias for the 6-OHDA-lesioned rats from the day 1 post-surgery. Both tests detected the motor asymmetry induced by (single and repeated) apomorphine (APO) challenges that correlated, in the first week, with the DAergic neuronal degeneration. The described TSST is fast and easy to perform, and in the drug-free condition is useful for the functional assessment of early motor asymmetry appearing after the 6-OHDA-lesion in the SNc, without the confounding effect of APO challenges.

## 1. Introduction

Parkinson’s disease (PD) is a neurodegenerative disorder characterized by the progressive loss of dopaminergic (DAergic) neurons in the substantia nigra pars compacta (SNc) [1,2,3]. The main treatment for PD is only symptomatic, aiming at increasing DAergic function in the striatum (ST) and improving the motor symptoms such as bradykinesia, rigidity, resting tremor, and postural instability [4].

Several animal models have been employed in experimental studies concerning the pathogenetic mechanism of PD and therapeutic strategies [5]. The classical unilateral 6-hydroxydopamine (6-OHDA) model, showing PD-associated pathological and behavioral features, is considered one of the most useful tools in PD research [6,7,8,9,10]. Over the years, to lesion the mesencephalic dopamine (DA) neurons and reproduce, to some degree, PD motor impairments, different concentrations of 6-OHDA have been infused either into the SNc, medial forebrain bundle (MFB), or the ST, leading to partial or complete neurodegeneration [7,8,10,11,12,13]. The doses of 6-OHDA used (from 4 to 24 µg) and the observation window (from 12 h to months) differ among studies. Boix et al. [14] investigated the consequences of different doses of 6-OHDA in the MFB of mice by detecting dose-dependent motor impairments and nigrostriatal cell and fiber loss 21 days after the surgery when mice achieved a complete post-lesion recovery. They observed that 6-OHDA doses of 0.7 μg and 1 μg caused partial denervation of the nigrostriatal fibers. Truong et al. [15] created a similar model in rats and tested the effects of different 6-OHDA doses on the lesion size by determining the percentage of cell loss and performing different behavioral tests between 1 and 10 weeks post-lesion. Specifically, they tested the effects of 4, 8, and 16 μg injections in the MFB and observed increasing cell loss and concomitant behavioral changes in the three models [15]. The striatal injection should therefore not be proposed as a progressive model of PD, but rather as one of the suitable early-stage models, thus useful for testing the efficacy of novel drugs [16].

The behavioral effects of 6-OHDA lesion are dependent on the above variables, which couple with the animal spontaneous recover capability over time. When delivered to the ST, 6-OHDA induces a slow, progressive, and partial damage to the nigrostriatal structure in a retrograde manner, for 3 weeks post-lesion [7]. A near-complete lesion is achieved by the administration of 6-OHDA (12 μg) to the MFB, the axonal projection pathway of the nigrostriatal system [7]. In the classical work by Jeon at al. [17], 8 μg of 6-OHDA in the SNc induced the degeneration of DAergic neurons starting as early as 12 h and produced after 2–3 weeks a maximal and stable lesion (>80% DAergic cell death in the SNc). The characterization of the lesion development following 6-OHDA infusion in the SNc is very important for small-size lesions, where compensatory mechanisms may act in a very effective way, and medium-size lesions, that need some time to fully develop into the chronic state [18,19]. Large-size lesions develop in a very short time, making the characterization of the transient phase very hard.

Most of the preclinical experiments have been focused on late-phase, chronic, fully DA-depleted states and it has been shown that behavioral motor biases are associated with a complete loss of the nigrostriatal pathway [6,8,10,12,14,15,16,18]. Indeed, only a few studies have focused specifically on the early-phase behavioral responses after the 6-OHDA lesion in the ST [7,10,20,21], MFB [7,13,22], and SNc [23,24]. It was reported that the ST lesion induces spontaneous motor impairments which are observed as early as the first day after lesion [20]. To date, the earliest behavioral characterization after the 6-OHDA lesion in rats has been reported in the ST and MFB, and it was shown that spontaneous motor impairments are observed as early as the first day after ST lesion [20]. The paucity of literature investigating the first week after the SNc lesion for the emergence of behavioral impairments in the 6-OHDA model prompted us to directly compare the onset and progression of the nigrostriatal degeneration and motor impairments during this phase.

Moreover, since PD cannot be prevented and neuroprotective therapy is at its infancy stage [25,26], we can anticipate the need for behavioral and diagnostic imaging tools capable to validate prospective treatments also in the early phase of the disease, when substantial numbers of DAergic neurons are still alive and treatments could be more effective [27]. Therefore, it is crucial to increase our understanding of the early pathophysiological mechanisms that occur after a toxin insult at the level of DAergic function. It might be interesting to study the early time-course (i.e., the first week) molecular and functional changes occurring in the emergence of the Parkinsonian lesion in the standard 6-OHDA model and whether they might be predictive of the severity of the lesion.

With this aim, we directly compared the onset and progression of motor impairment bias and its correlation with the severity of the nigrostriatal degeneration up to three weeks after the injection of 6-OHDA. To determine the emergence over time of potential spontaneous motor deficits, we modified the tail suspension test [28,29], here indicated as tail suspension swing test (TSST). We compared the TSST results to those obtained with the standard rotation test (RT) [30]. Our findings show that the TSST can be more effective for the spontaneous motor bias characterization of the earliest pathophysiological mechanisms and, eventually, be useful for testing new neuroprotective strategies.

## 2. Results

### 2.1. Study Design

We investigated whether early motor behavioral deficits can be observed in the first week after the injection of 8 μg of 6-OHDA into the right SNc. Moreover, we compared the developing motor behavioral deficits, the nigral cells and striatal fibers loss over three weeks post-lesion (days 1, 3, 5, 7, 14, 21). Thus, four groups of animals were evaluated over time: 6-OHDA-lesioned rats (6-OHDA) and sham rats (SHAM), each with the two subgroups, single apomorphine (APO) administration (*n* = 23 6-OHDA-S; *n* = 14 SHAM-S) and repeated APO administration (*n* = 18 6-OHDA-R; *n* = 12 SHAM-R).

The animals were randomly assigned to the SHAM and the 6-OHDA-lesioned groups. The experimental protocol was the following: pre-surgery (day 0), all rats (*n* = 67) were tested in drug-free condition first with TSST (200 s), followed by RT (10 min); stereotactic surgery (day 0); all testing days post-lesion the rats were tested in drug-free condition first with TSST (200 s), followed by RT (10 min). We aimed to test whether the administration of APO (0.5 mg/kg in 0.9% saline, s.c.) could modify the TSST swing results. Also, we assessed the TSST capability, following APO challenge, to evaluate the extent of DAergic loss in the SNc as well as terminal fibers loss in the ST. For comparison, the standard RT was also performed. Thus, we selected the 6-OHDA-S and SHAM-S subgroups (*n* = 37), receiving a single APO administration, at 1 or 3 or 5 or 7 or 14 or 21-days post-lesion. On the testing day, the animals were first assessed in drug-free condition with the TSST (200 sec) followed by RT (10 min), and after APO administration the rats belonging to the 6-OHDA-S subgroup were tested first with TSST (200 s), followed by RT (60 min).

Moreover, to study possible sensitization effects due to the repeated APO treatment (every single dose 0.5 mg/kg in 0.9% saline, s.c.), at day one post-lesion, we selected the 6-OHDA-R and SHAM-R subgroups (*n* = 30), receiving multiple APO administrations at (1, 3) (1, 3, 5), (1, 3, 5, 7), (1, 3, 5, 7, 14), or (1, 3, 5, 7, 14, 21) days post-lesion, with the sacrifice on the last testing day. On each of these testing days, the rats of the 6-OHDA-R subgroup were first tested in drug-free conditions with TSST (200 s), followed by RT (10 min) and, after APO administration, tested with TSST (200 s), followed by RT (60 min). It is worth noting that, in this protocol, at day 1 post-lesion all the rats belonged to the 6-OHDA-S (or SHAM-S) subgroup. All animals were sacrificed, the rat brains were fixed and analyzed with histology and immunohistochemistry (IHC).

### 2.2. Behavioral Results

#### 2.2.1. Tail Suspension Swing Test

**Figure 1:**
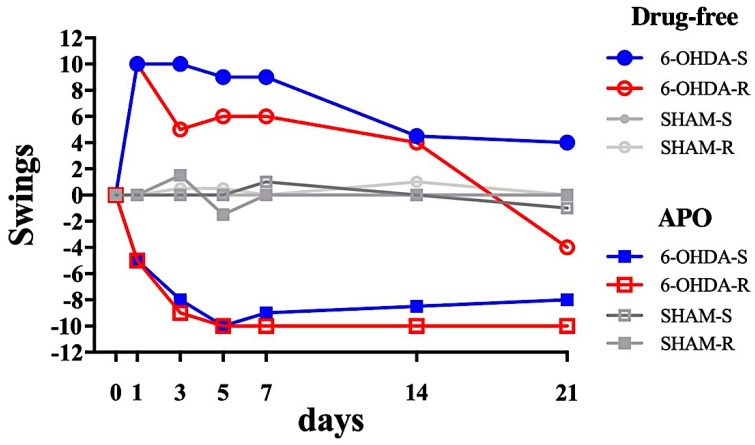
Time course of the number of swings assessed by the tail suspension swing test (TSST) for the SHAM and 6-OHDA lesioned rats in drug-free (circles) and apomorphine (APO, squares) induced condition. Day 0 represents the pre-surgery testing. 6-OHDA-S (blue) and 6-OHDA-R (red) are rats subjected to single (on the sacrifice day) or repeated (all testing days until sacrifice day) APO administration, respectively. Positive values indicate ipsilateral swings; negative values indicate contralateral swings. See Table S1 for the statistical difference between 6-OHDA and SHAM groups, and between time points to the pre-surgery condition (day 0).

The temporal evolution of the swings (the difference between the ipsilateral and the contralateral swings), in drug-free and after APO administration in SHAM and 6-OHDA lesioned rats is shown in Figure 1. The whole data set is reported in Table S1.

In the drug-free condition, both 6-OHDA-S and 6-OHDA-R rats showed a high positive number of swings, representing a movement to the ipsilesional side (Figure 1). The number of swings of the SHAM rats remained, as for the pre-surgery result, close to zero. For the 6-OHDA-S rats, the Kruskal-Wallis test revealed a significant effect of lesion treatment (*p* < 0.05). Dunn’s multiple comparisons post-hoc test showed significant differences between SHAM-S and 6-OHDA-S on days 1, 3, 5, and 7 (*p* < 0.05), absent on days 14 and 21 (*p* > 0.99). There was also a significant variation between time points in the 6-OHDA-S rats (*p* < 0.05 for days 1 through 5 versus pre-surgery), absent in the SHAM-S rats. For the 6-OHDA-R rats, the Kruskal-Wallis test revealed a significant lesion effect (*p* < 0.05). Dunn’s post-hoc showed significant differences between SHAM-R and 6-OHDA-R on days 3, 5, and 7 (*p* < 0.05), but not on days 14 and 21 (*p* > 0.99). There was also a significant variation between time points in the 6-OHDA-R group (*p* < 0.05 for days 3 through 7 versus pre-surgery), absent in the SHAM-R group.

In the APO-induced condition, both 6-OHDA-S and 6-OHDA-R rats showed a high negative number of swings, indicating movement to the contralesional side (Figure 1) since the first-day post-lesion. The number of SHAM swings remained close to zero. For the 6-OHDA-S animals, the Kruskal-Wallis test revealed a significant lesion effect (*p* < 0.05). Dunn’s multiple comparisons post-hoc test showed significant differences between SHAM-S and 6-OHDA-S on all days (days 1 through to 21, *p* < 0.05). There was also a significant variation between time points in the 6-OHDA-S rats (*p* < 0.05 for days 1, 7, 14, 21 versus pre-surgery), absent in the SHAM-S group. For the 6-OHDA-R animals, the Kruskal-Wallis test revealed a significant lesion effect (*p* < 0.05). Dunn’s post-hoc showed significant differences between SHAM and 6-OHDA-R on all days (days 1 through to 21, *p* < 0.05). There was also a significant variation between time points in the 6-OHDAR rats (*p* < 0.05 for all days versus pre-surgery), absent in the SHAM. For the 6-OHDA-R rats, there was a gradual increase of the contralateral swing bias during the first 5 days after the lesion, followed by a plateau from 7 to 21 days after the lesion. The 6-OHDA-S rats showed a similar swing trend in the first 5 days post-lesion, although the plateau values are lower compared to the 6-OHDA-R rats’ ones.

#### 2.2.2. Rotational Test

**Figure 2:**
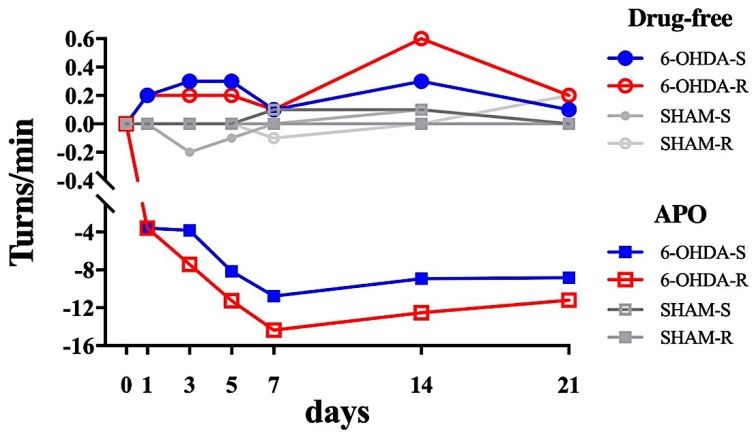
Time course of the number of turns/min assessed by the rotational test (RT) for the SHAM and 6-OHDA lesioned rats in drug-free (circles) and apomorphine (APO, squares) induced condition. Day 0 represents the pre-surgery testing. Positive values indicate ipsilateral turns/min; negative values indicate contralateral turns/min. See Supplementary Table S2 for the statistical difference between 6-OHDA and SHAM groups, and between time points to the pre-surgery condition (day 0).

The temporal evolution of the rotational response (the difference between the ipsilateral and contralateral rotations, expressed as the number of turns/min) in drug-free and after APO administration in SHAM and 6-OHDA lesioned is shown in Figure 2. The whole data set is reported in Table S2.

In the drug-free condition, both 6-OHDA-S and 6-OHDA-R rats showed a positive and very small number of turns/min, indicating a limited tendency to rotate toward the ipsilesional side (Figure 2). The number of turns/min of the SHAM groups remained close to zero (Figure 2). For both 6-OHDA-S and 6-OHDA-R rats the Kruskal-Wallis test showed a significant lesion effect between SHAM and 6-OHDA-lesioned rats only on day 1 post-lesion (*p* < 0.05, Dunn’s post-hoc). For the 6-OHDA-S rats, Dunn’s post-hoc analysis showed significant differences of turns/min between time points in the 6-OHDA-S rats (*p* < 0.05 for days 1 and 14 versus pre-surgery), but not in the SHAM group. For the 6-OHDA-R rats, Dunn’s post-hoc analysis revealed a significant difference of turns/min between time points in the 6-OHDA-R rats (*p* < 0.05 for days 3, 5, 14 versus pre-surgery), but not in the SHAM group.

In the APO-induced condition, both 6-OHDA-S and 6-OHDA-R rats showed a negative high number of turns/min, indicating movement toward the contralesional side (Figure 2). The number of turns/min of the SHAM groups remained close to zero. For the 6-OHDA-S rats, the Kruskal-Wallis test showed no significant main effect of the factor treatment between SHAM and 6-OHDA-lesioned rats on all days. Dunn’s post-hoc analysis showed significant differences of swings between time points in the 6-OHDA-S animals (*p* < 0.05 for days 1, 7, 14, 21 versus pre-surgery), but not in the SHAM group. For the 6-OHDA-R rats, the Kruskal-Wallis test revealed a significant main effect of the factor treatment (*p* < 0.05). Dunn’s post-hoc showed significant differences between SHAM and 6-OHDA-R on all days (days 1 through 21, *p* < 0.05). There was also a significant difference between time points in the 6-OHDA-R rats (*p* < 0.05 for all days versus pre-surgery), absent in the SHAM group. For the 6-OHDA-R animals, there was a gradual increase in the contralateral bias during the first 5 days after the lesion, followed by a plateau from 7 to 21 days after the lesion. The 6-OHDA-S rats showed a similar trend in the first 5 days post-lesion, although the values at the plateau were lower compared to the 6-OHDA-R ones (Figure 2).

### 2.3. Immunohistochemical Results

**Figure 3:**
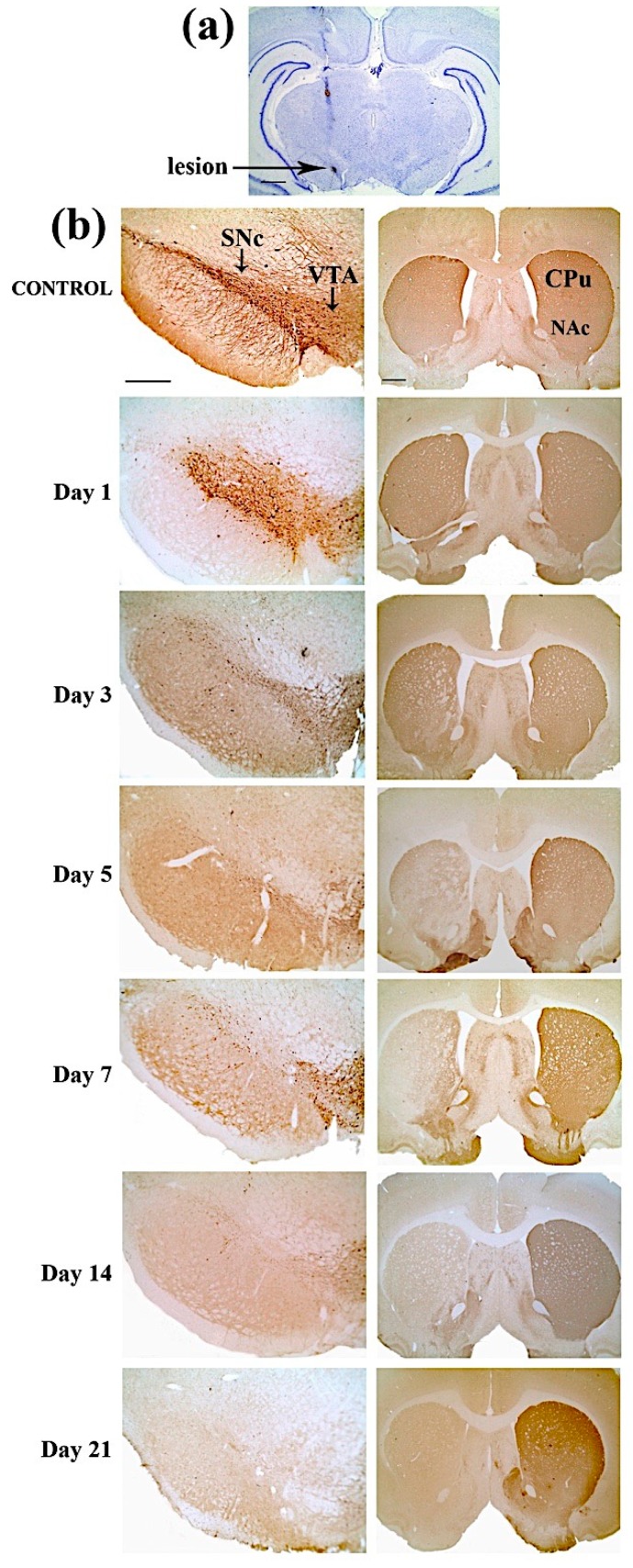
(**a**) Cresyl violet staining of a representative coronal brain section (the arrow indicates the lesion site in the right SNc). Scale bar: 1 mm. (**b**) Time course, for each time point from day 1 to 21 post-lesion, of representative coronal sections for Tyrosine Hydroxylase-immunoreactive (TH-ir) nigral cell (left column) and TH-ir striatal fibers loss (right column) in control (first row) and 6-OHDA lesioned animals. Abbreviations: SNc (Substantia Nigra compacta); VTA (Ventral Tegmental Area); CPu (Caudate Putamen); and NAc (Nucleus Accumbens). Scale bar: 500 µm (left column), 1 mm (right column).

Figure 3a shows the cresyl violet staining of a representative coronal section with a trace of the microsyringe trajectory. The staining confirmed that the lesion was correctly placed in the right SNc. Typical examples of TH-immunostained sections used to quantify the effects of the lesion, measuring the differences in TH immunoreactive (TH-ir) striatal fibers and SNc cell counting among ipsilateral and contralateral cerebral hemispheres, are shown in Figure 3b. The control rats did not show TH-ir cells and fibers asymmetry in the striatum and the SNc, respectively, as shown in Figure 3b.

As shown in Figure 3b, a prominent loss of TH-ir cells was present in the SNc and a reduced effect was also visible in the VTA. This reflects the surviving TH-ir innervation of the ventral ST observed during the first week after lesion. At 21 days after lesion, a complete loss of DAergic neurons, notably both in the SNc and the VTA, and a near-complete loss of DAergic innervation of the ST were observed. Thus, our results are in agreement with the observation that SNc projects to the dorsal ST, and the VTA to the ventral ST [31].

**Figure 4:**
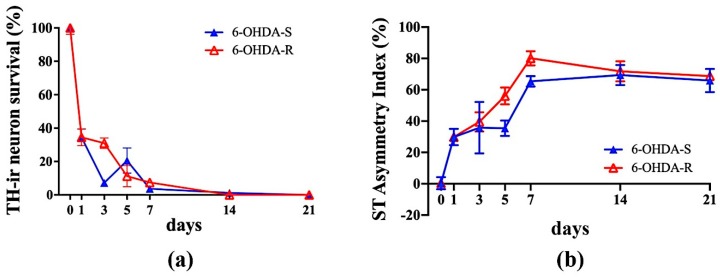
(**a**) Time course of the TH-ir neuronal loss in the SNc of the 6-OHDA lesioned animals subjected to single (6-OHDA-S, filled triangle) or repeated (6-OHDA-R, open triangle) apomorphine administration. (**b**) Time course of TH-ir terminal fiber percentage asymmetry index in the Striatum (ST) of 6-OHDA-S (filled triangle) and 6-OHDA-R (open triangle) lesioned animals.

A progressive loss of TH-ir neurons in the SNc was observed in the 6-OHDA lesioned animals over the post-lesion observation time, with an almost total loss practically reached on day 7 (Figure 4a). The 6-OHDA-S rats showed a rapid decrease of DA cells in SNc. In contrast, DA cell loss was much slower in 6-OHDA-R. Tukey’s HSD post-hoc comparison reached the level of significance (*p* < 0.05) when comparing: 6-OHDA-S, one day after the lesion vs. pre-surgery condition (day 0); 6-OHDA-S, one day after the lesion vs. all days except 5 days; 6-OHDA-R: 3 vs. 21 days after the lesion. Consistently a progressive increase in the striatal TH-ir terminal fibers asymmetry index of 6-OHDA lesioned rats was observed over the first-week post-lesion, with the plateau already reached on day 7 (Figure 4b). Tukey’s HSD post-hoc comparison reached the level of significance (*p* < 0.05) when comparing: 6-OHDA-S, one day after the lesion vs. pre-surgery condition (day 0); 6-OHDA-S, one day after the lesion vs. 7, 14, and 21 days; 6-OHDA-R: 3 days vs. 7 through 21 days.

### 2.4. Regression Analysis

**Figure 5:**
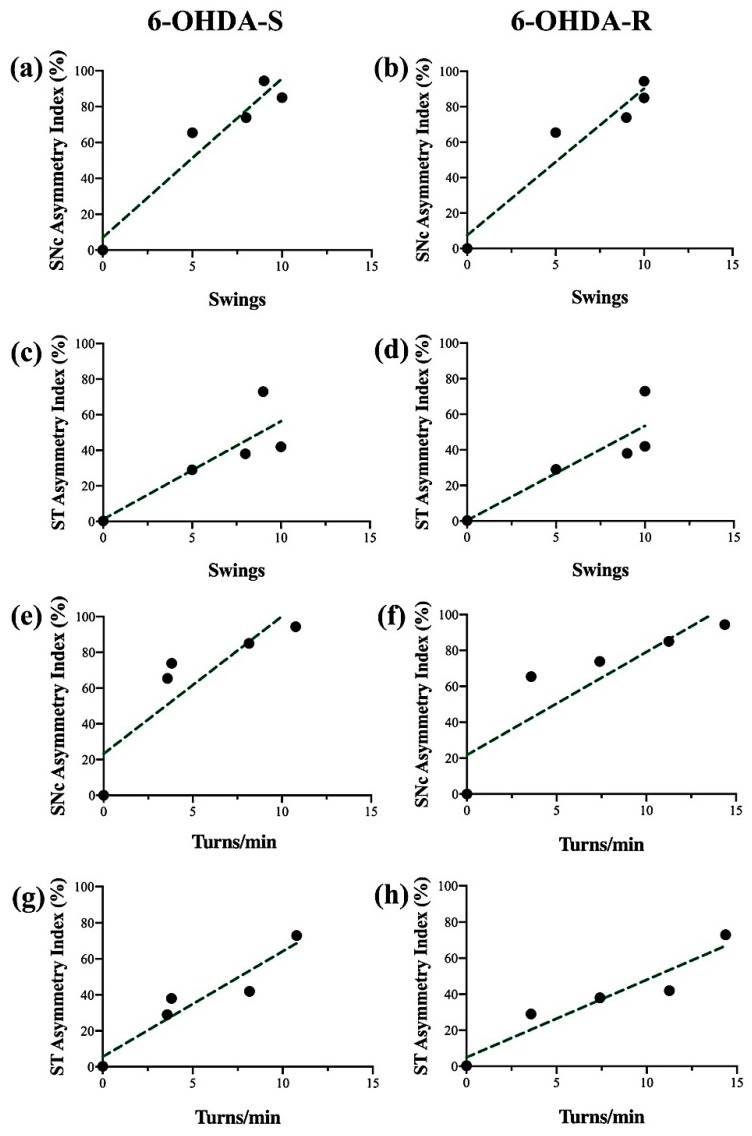
Regression analysis of contralateral TSST swings (**a**–**d**) and RT turns/min (**e**–**h**) versus the SNc asymmetry index % nigral cell loss (**a**,**b**,**e**,**f**) and ST asymmetry index % terminal fiber loss (**c**,**d**,**g**,**h**) for the animals subjected to single (6-OHDA-S, left column) and repeated (6-OHDA-R, right column) apomorphine administration.

A regression analysis related to the first-week post-surgery was performed, discarding the plateau phase (days 14 and 21). Figure 5 shows the performance of the APO-treated 6-OHDA-lesioned rats in the TSST (a through d) and RT (e through h), compared with the percentage asymmetry index of the SNc (a, b, e, f) and ST (c, d, g, h) for both 6-OHDA-S and 6-OHDA-R rats. The analysis showed that the TSST had a predictive value for both TH-ir nigral cell loss (6-OHDA-S: R = 0.92, slope = 8.86, F = 35.34; 6-OHDA-R: R = 0.92, slope = 8.27, F = 34.91) and striatal denervation (6-OHDA-S: R = 0.72, slope = 5.50, F = 7.89; 6-OHDA-R: R = 0.77, slope = 5.31, F = 10.20). The contralateral APO-induced rotations assessed by the RT correlate with the loss of nigral TH-ir neurons (e, f) (6-OHDA-S: R = 0.75, slope = 7.68, F = 9.38; 6-OHDA-R: R = 0.78, slope = 5.73, F = 10.91) and the striatal percentage asymmetry index (g, h) (6-OHDA-S: R = 0.89, slope = 5.86, F = 26.07; 6-OHDA-R: R = 0.90, slope = 4.3, F = 27.91). There was no correlation between the two behavioral tests in drug-free conditions and the extent of TH-ir striatal denervation or nigral cell loss (data not shown).

## 3. Discussion

In the present study, we report the first three-weeks behavioral characterization of the effects of unilateral 6-OHDA SNc lesion in rats, showing that our newly developed TSST method in drug-free conditions allows revealing the presence of an ipsilateral swinging asymmetry as early as the first-day post-lesion. Our finding is in agreement with early evidence indicating that severe nigral lesioned 6-OHDA rats show from day 1 a spontaneous ipsilateral turning and scanning behavior [23], while these ipsiversive asymmetries become apparent after the first week in striatal-lesioned animals [32].

So far, only a few studies have focused on the early-phase nigrostriatal degeneration and spontaneous motor deficits in the SNc 6-OHDA model [23,24,33]. Indeed, it is common to wait three weeks after surgery for a stable nigrostriatal lesion development before using the lesioned animals for further analyses [8,34,35,36,37]. The most prominent deficits reported for the 6-OHDA-injected models are asymmetries in sensorimotor reactivity starting from 6 weeks [8], posture over 19 weeks [35], impairments of motor coordination and balance skills starting from 2 weeks [10] after lesion. In a recent study, combining PET imaging and Catwalk gait analysis 4 weeks after 6-OHDA lesion in the MFB, it was shown that altered brain metabolism and gait abnormalities correlate with DAergic lesion severity in the rat, indicating that striatal DA depletion, focal changes of glucose metabolism, and motor impairments are strongly linked [38].

Considering that the currently available PD treatments are more effective in early diagnosed patients [25,26,27], there is a need to study the whole range of animal models that may help in the characterization of the early pathophysiological mechanisms underlying PD. Considering the current literature on pre-clinical PD models, we focused our behavioral and structural study on early pathophysiological mechanisms occurring after a toxin insult at the level of DAergic function, adopting the classical SNc 6-OHDA-lesioned model. To this purpose, we aimed to develop a novel behavioral method capable to provide a sensitive response to the early behavioral changes occurring after the toxin injection both in the drug-free condition and after single and multiple APO administration. We found that the biased swinging behavior observed in 6-OHDA-lesioned rats subjected to TSST can serve as a sensitive indicator of DAergic neurodegeneration. Indeed, in drug-free conditions, the TSST allowed detecting a motor asymmetry expressed as a stable ipsilateral swinging index of about nine swings from day 1 to day 7 in all the 6-OHDA-S animals. At weeks 2 and 3 post-lesion the drug-free TSST response showed a gradual decrease toward baseline. In drug-free conditions, the 6-OHDA-R animals showed a similar TSST response, although the maximum swinging response at days 3, 5, 7 was of minor amplitude (about 6 swings). In contrast, in drug-free conditions, the elevated body swing test (EBST) [28], from which we derived our TSST, revealed a contralateral swing at seven days post-lesion in the SNc 6-OHDA-lesioned rats. Other EBST studies confirmed contralateral biased swings in drug-free conditions following intrastriatal 6-OHDA lesion [39,40], accordingly with the direction of APO-induced rotational behavior. These authors suggested that the direction of APO-induced rotational behavior is rather dependent on the location (anterior/posterior coordinates) of neurotoxin injection into the striatum. A similar location effect dependence could be expected for the TSST versus EBST swing bias asymmetry in the SNc 6-OHDA-lesioned rats. Another possible explanation for the inconsistency between the TSST (ipsilateral) and EBST (contralateral) drug-free results might be due to the different experimental approaches with the EBST causing a higher level of stress to the animal by suspending it from its tail continuously for 60 sec. Stress is indeed capable of altering DA function and locomotor activity [41]. Our TSST-modified experimental procedure, instead, decreased the suspension time to a maximum of 10 sec per trial and likely it reduced the associated stress. The swing behavior is clearly due to an asymmetry in motor performance since SHAM animals did not show it, and it might be triggered by DA release from the intact side, causing a swing in the ipsilateral direction [42]. An alteration of involuntary reflexes, such as the vestibular-driven response might contribute to the swings. The number of swings in the drug-free state halved at 14 and 21 days after the lesion. The reason for this decreased sensitivity is difficult to elucidate. It might be related to plastic changes and/or recovery of different systems after two-three weeks post-lesion. A possible explanation may be concerned with the establishment of compensatory mechanisms within residual neurons in the lesioned side [8,18,43] and the recovery could depend on movement strategies based on the ability of animals to compensate for their postural bias [44]. Recently, the importance of gait compensatory mechanisms for the MFB 6-OHDA-lesioned rats was correlated with DAergic lesion severity [38], providing support to the previous hypothesis.

Interestingly, the repeated APO treatment (6-OHDA-R rats) received on the day(s) before the TSST tended to reduce the drug-free swing response reaching a significant effect on day 21. This shows that APO administration the days before testing activates mechanisms opposing the drug-free swinging, and this effect may be due to a decrease in DA receptors, induced by repeated APO administration, or to receptor desensitization changes in the intact striatum [30,43,44,45,46,47,48,49,50,51,52,53,54]. On the other hand, in our study when APO was administered before the TSST, it induced contralateral swings in 6-OHDA animals with the same direction bias to that obtained using EBST [28], i.e., away from the lesioned side. This biased swing direction is likely due to the APO action at the post-synaptic level and results in the hyperstimulation of supersensitive DA receptors in the denervated striatum [30]. This evidence further supports the possibility that our TSST in a drug-free state engages different brain circuits, while the EBST does not show a spontaneous contralateral swing, rather a bias induced by contralateral DA activation (possibly stress-mediated).

The contralateral swinging of the animals subjected to TSST, both after a single or repeated APO administration, increased in the first-week post-lesion, reaching a plateau at 7 days; this probably is caused by the increase of the DA receptor hypersensitivity as a result of the progression of the striatal lesion [30]. The APO swinging scores correlated with nigral DA cell loss and TH-ir striatal fibers degeneration between day 1 and day 7, and at 14 and 21 days post-lesion reached the maximum plateau level. It is worth noting that at the later times (days 7, 14, and 21) the rats subjected to repeated APO administration showed a slightly greater swing than the ones with a single APO dose (10 vs. 8.5 swings), although the difference was not statistically significant. In agreement with this evidence, the RT results from 3 to 21 days post-lesion, in drug-induced condition, showed that the rats subjected to the repeated APO administration showed a greater rotation bias than the ones receiving a single APO dose (21 days: 11.2 vs. 8.8 turns/min), although the difference was not statistically significant. Our interpretation of these findings is that the systematic behavioral difference, in the swing and rotational plateau values, between rats subjected to single or multiple APO administration may derive from the development and maintenance over time of DAergic postsynaptic receptors supersensitivity. However, future work is required to validate this hypothesis.

Much work has been devoted to analyzing the turning behavior after systemic treatment with APO and how responses develop over time [52,55]. We revealed, as the majority of the studies [23,24], that the contralateral turning response to APO increases from the first day up to one week after lesion and correlates with the lesion severity progression [15,35,53,54]. The increased turning response is due to the hypersensitivity of the lesioned side of the brain while the contralateral one remains normo-sensitive. The dopamine D1/D2 agonist APO increases motor activity by directly stimulating the post-synaptic DA receptors of the striatum [56,57]. A new finding of the current study is that APO treatment for both the single and repeated APO challenges progressively increased TSST contralateral swings behavior during the first five days following the lesion, although tolerance to APO treatment has also been described [56,57]. The TSST results in APO-induced testing showed that the 6-OHDA-lesioned rats subjected to single and repeated APO administration had a significantly different response compared to the SHAM animals over time (1 to 21 days post-lesion). While only the 6-OHDA-lesioned rats subjected to repeated APO administration showed an RT response significantly different compared to the SHAM animals at all times.

With the dose of 6-OHDA used here (8 µg), we observed at day 1 post-lesion about 65% neuronal loss in SNc and about 30% in striatal terminal fibers in agreement with other studies [17]. The lesion reached its maximum at 14 days for DA cell and 7 days for the striatal TH-ir asymmetry index, in agreement with a previous study [23] but in contrast with others that reported a marked loss of striatal terminals established within 2-3 days post-lesion [6,58]. Here, we found that the maximum peak of contralateral turns was reached five days post-lesion, corresponding to a DA depletion of about 90%. In agreement with this finding, was reported that the drugs’ ability to induce contraversive turning is dependent on DA depletion and becomes most evident when it exceeds about 95% [59]. Finally, the correlation between behavior asymmetry and the lesion was observed only in APO-induced swinging and rotation but not in spontaneous condition, which shows a considerable recovery within the last two weeks after lesion. Indeed, there was no correlation between the two behavioral tests in drug-free conditions, the extent of TH-ir striatal denervation and cell loss.

Our TSST swing data show a strong behavioral effect already on day 1 after the 6-OHDA lesion in the SNc. The TH-ir neuronal loss on day 1 was about 65%, reaching the level of 95% within 7 days post-lesion. The brain operation trauma in the first 24 h post-lesion may have caused some degree of bias in the measured behavioral swing and rotation responses. In principle, this infusion-related mechanical damage bias on our behavioral data cannot be ruled out. However, if this effect was present it would equally affect both the 6-OHDA and SHAM animals, while not altering inter-groups comparison. Thus, the above-hypothesized bias condition, along the full three-weeks observation time, is not consistent with our behavioral data, consequently, we can assume that the TSST and RT results reported here are dependent on the specific toxin effect only. The described TSST in drug-free conditions allows identifying well-lesioned animals already at 1 day after the toxin infusion [33]. This result is of particular importance given that the behavioral changes occurring during the onset of neurodegeneration following 6-OHDA lesions have been poorly characterized in the literature. Our study aimed to fill this research gap by providing behavioral and immunohistochemical data in the first week after the SNc 6-OHDA lesion, which may be of help in understanding the early pathophysiological mechanisms in PD.

## 4. Material and Methods

### 4.1. Animals and Surgical Procedure

Adult male Sprague-Dawley rats (Envigo, San Pietro al Natisone, Italy) (*n* = 72) weighing 250–350 gr were randomly assigned to the SHAM-lesioned (*n* = 26), 6-OHDA-lesioned (*n* = 41), and control (*n* = 5) groups. All animals were individually housed in a temperature-controlled room under an inverted light/dark cycle with free access to tap water and standard food. The experimental protocol for animal care and handling was according to the European Community Council Directive (86/609/EEC) and the Italian law n. 26 (14/03/2014) and was approved by the Institutional Review Board of the University of L’Aquila. A subset of experiments was performed at the Department of Physiology and Biochemistry at the University of Malta in accordance with 86/609/EEC and national Institutional Animal Use and Care Committee (IAUCC) directives of the University of Malta. All animals were anesthetized with ketamine (80 mg/kg, i.p.) and xylazine (0.5 mg/kg, i.p.) before surgery.

In the PD animal model studies, a wide range of 6-OHDA doses (1.6 to 24 μg) and infusion volumes (0.5 to 4 μL) have been reported. In this study, we used one of the most commonly adopted SNc 6-OHDA lesioning protocols, [17,30,60,61,62,63,64,65,66]. Rats were lesioned in the right SNc according to the Paxinos and Watson’s atlas [67] at coordinates (+5.40 mm posterior to Bregma;−2.10 mm lateral to the midline and +7.40 mm below the dura) by infusing 6-OHDA (Sigma Aldrich, MO, USA) (8 µg/4 µL) dissolved in saline containing ascorbic acid (0.1%) (vehicle solution). A volume of 4 µL was injected over 5 min, leaving the microsyringe in situ for further 5 min. SHAM-operated animals were subjected to the same surgical procedure but receiving buffered saline solution only.

For IHC analysis: (i) Five animals did not receive any surgical procedure and were sacrificed as the control group (day 0); (ii) at each time point from 1 to 21 days, a total of five 6-OHDA-lesioned animals were used. During surgery and recovery, animals were kept warm using a heating pad. All animals did not receive pain drugs (such an analgesic) to avoid interference on neurotransmitter levels and toxicity-induced effects [68,69]. After surgery, the animals were monitored until their awakening and checked during the subsequent days of the experimental protocol.

### 4.2. Behavioral Tests

The behavioral parameters were collected before surgery (day 0), and at 1, 3, 5, 7, 14, 21 days post-lesion. On the testing day, the observations were carried out from 09:00 to 12:00 a.m. To minimize possible changes derived from environmental influences, the rats were left to acclimate in the procedure room for 30 min before the test. As described in the study design, the rats were assessed first in the drug-free condition (TSST: 200 sec; RT: 10 min), and then tested (TSST: 200 sec; RT: 60 min) after APO challenge (0.5 mg/kg in 0.9% saline, s.c.). This APO dose has been used in previous studies of 6-OHDA SNc lesion for behavioral characterization [13,30,64,70].

#### 4.2.1. Tail Suspension Swing Test (TSST)

**Figure 6:**
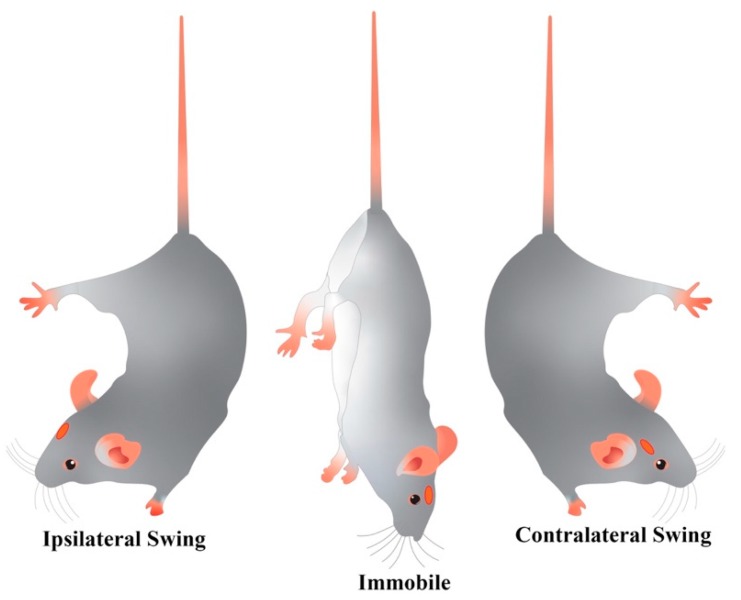
Drawing representing the swinging behavior (ipsilateral or contralateral deviation from animal vertical axis) evaluated with the tail suspension swing test (TSST) in 6-OHDA rats with a lesion on the right SNc (red dot).

Our newly developed TSST comprised ten trials of a maximum of 10 sec, each followed by 10-sec rest. The rat tail was taken approximately 3 cm from its base, the animal was elevated to about 5 cm above the cage floor and held along the vertical axis for 10 sec. Some animals had the tendency to climb their tail, so this experimental procedure was drawn up to avoid all the effects derived from the stress caused by handling the rat by its tail for a long time, i.e., 60 sec continuously as reported in previous literature [71,72]. This downright position was considered stable if no swing to either the left or the right head side was observed. A positive (ipsilateral, I) or negative (contralateral, C) swing was counted whenever the animal moved its head out of the vertical axis to either side by at least 10° within the 10-s observation interval, as shown in Figure 6, and then gently and immediately positioned on the floor. Only the first swing (if any) was counted. Then the animal was placed back on the ground for 10 sec, with the four paws on the floor, before the beginning of the next trial. Swings were recorded using a hand counter. The TSST behavioral index was obtained from the 10-trial set as a net number of swings (I—C).

#### 4.2.2. Rotation Test (RT)

The RT was conducted in a home-made open field apparatus [73] consisting of a transparent Plexiglass box (50 cm × 50 cm × 50 cm) with a floor of opaque black material illuminated by a dim white light located above the arena. The arena was carefully cleaned before each test. Rats were placed at the center of the arena and allowed to freely explore the environment for 10 min, under baseline condition APO-free. Finally, the rats were tested for rotational behavior for 60 min following APO administration. The rotational data were obtained by observations of stereotyped behaviors by an expert operator in blind. Each RT session was also videotaped (HD movies recorded at 30 fps) for subsequent automatic behavioral analysis.

### 4.3. Tissue Processing

At the end of the behavioral session, the rats were deeply anesthetized with chloral hydrate (400 mg/kg i.p.) and transcardially perfused with 100 ml of heparinized saline, followed by perfusion with 300 mL of 4% paraformaldehyde in 0.1 M phosphate buffer (pH 7.4) (Panreac Quimica, Barcelona, Spain). The brains were carefully removed and stored in the same fixative solution for at least 160 h at 4 °C to allow a full fixation process. The brains were then immersed in a cryoprotective solution of 15% and 30% sucrose for 72 h and then cut at 30 µm with a sliding microtome (Cryomat 1700, Leitz, Wetzlar, Germany). The sections were processed for histological (Cresyl Violet, CV) and TH-ir analyses.

### 4.4. Immunohistochemistry

The sections were rinsed three times in potassium phosphate-buffered saline (KPBS) and then endogenous peroxidase activity was quenched in 0.3% H_2_O_2_ and absolute methanol. Subsequently, the sections were incubated in a solution consisting of 5% normal goat serum in PBS and 0.25% Triton X-100, to block nonspecific binding sites and then incubated overnight at room temperature in the same blocking solution as described above with the mouse anti-tyrosine hydroxylase (TH; 1: 1000; AB152; Chemicon, Merck Life Science S.r.l., Milano, Italy) as the primary antibody. On the second day, the sections were rinsed three times with PBS and then incubated in a 1:200 dilution of the biotinylated secondary antibody, goat anti-mouse (Vector Laboratories L.t.d., Peterborough, United Kingdom). The sections were treated with avidin-biotin–peroxidase-complex (ABC Elite kit; Vector Laboratories). Immunolabeling was revealed incubating the sections in 3.3%-diaminobenzidine (DAB) as the chromogen (peroxidase substrate KIT-DAB, Vector Laboratories L.t.d., Peterborough, UK). The sections were dehydrated in a gradual concentration of alcohol solutions (from 70% to 100%), cleared in xylene and cover-slipped using Canada balsam. Sections were examined on a Leica IM500 stereomicroscope acquiring RGB images (2048 × 1536 pixels; 96 dpi; 24 bit).

### 4.5. Quantitative Analysis

The number of TH-ir in the SNc was measured in both hemispheres using Cell Profiler [74]. In the ventral midbrain, TH-ir neurons of the intact and lesioned SNc were counted under brightfield illumination at 1× up to 20× magnification. Three sections along the rostrocaudal axis of the SNc (AP = −4.40; −5.40; −6.40 mm) were considered from each rat for cell counting. The TH-ir neuron survival index was calculated as the percentage number of TH-ir neurons in the ipsilateral side divided by the number of TH-ir neurons in the contralateral side. The extent of striatal denervation, as a consequence of lesion, was measured by optical densitometry considering three TH-stained sections (AP = 1.60; 0.70; −0.40 mm). The entire ST was divided into two halves along the dorsoventral axis and the measured values were corrected for nonspecific background staining by subtracting values obtained from the corpus callosum (ImageJ, version 1.32j; NIH, USA). Representative examples of the central striatal section are reported in Figure 3b. The density of TH-ir staining in the ST, given by the mean value of the DAB color signal, was quantified through the ImageJ system [75]. The ST TH-ir asymmetry index considers the Corpus Callosum (CC) as a reference signal intensity and is defined as:

[(signal Ipsi CC − signal Ipsi ST)/(signal Contra CC − signal Contra ST)] × 100

### 4.6. Statistical Comparisons

The TSST and RT behavioral data were analyzed using the non-parametric Kruskal-Wallis test and Dunn’s post-hoc. At any time post-lesion, only rats showing at least one swing (TSST: 200 s) in drug-free and at least one turn/min (RT: 60 min) in APO condition were included in the statistical analysis. The results are reported as the median and 25th/75th percentile values.

All the IHC data were expressed as mean value ± SEM. We used a multifactorial analysis of variance (ANOVA) with the group, time evolution and treatment as between factors. The Tukey’s HSD post-hoc comparison between means was used.

A level of *p* < 0.05 was considered to indicate a statistically significant difference. All statistical analyses were performed using Prism 8 GraphPad Software (San Diego, CA, USA).

## 5. Conclusions

An understanding of the mechanisms activated in the interval between the initial neurodegeneration and the late-stage motor symptoms is of great importance in the development of new early diagnostic methods and early therapies for PD. The described TSST is fast to perform, it does not require particular skills and in the drug-free condition developed in this study might be, therefore, useful for the functional assessment of early motor asymmetry appearing soon after the 6-OHDA-lesion in the SNc. Moreover, TSST offers an alternative tool for those studies on early-phase animal models of PD diseases that need to avoid the confounding effect of APO treatment.

## Supplementary Materials

Supplementary materials can be found at https://www.mdpi.com/1422-0067/21/8/2874/s1.
