# Supplementary material for: Comparison between Tail Suspension Swing Test and Standard Rotation Test in Revealing Early Motor Behavioral Changes and Neurodegeneration in 6-OHDA Hemiparkinsonian Rats
Source: Int J Mol Sci. 2020 Apr 20;21(8):2874. doi: 10.3390/ijms21082874 (PMC7216013; doi:10.3390/ijms21082874)
Supplement: Supplementary file 1 [file ijms-21-02874-s001.pdf]

Table S1. Summary of the swings assessed by the Tail Suspension Swing Test (TSST) before (day 0) and after (days 1 to 21) the lesion for the 6-OHDA and SHAM groups, in drug-free (FREE) and after apomorphine (APO) administration for the single (S) and repeated (R) apomorphine administration protocols.

**Days**	**6-OHDA**								**SHAM**							
	**FREE**				**APO**				**FREE**				**APO**			
	**S**	**25th<br>75th**	**R**	**25th<br>75th**	**S**	**25th<br>75th**	**R**	**25th<br>75th**	**S**	**25th<br>75th**	**R**	**25th<br>75th**	**S**	**25th<br>75th**	**R**	**25th<br>75th**
**0**	0.0	- 2.0<br>2.0	/	/<br>/	0.0	- 2.0<br>2.0	/	/<br>/	0.0	- 2.0<br>2.0	/	/<br>/	0.0	- 2.0<br>2.0	/	/<br>/
**1**	10.0$^{*, \#}$	8.0<br>10.0	/	/<br>/	- 5.0$^{*, \#}$	- 7.0<br>- 4.0	/	/<br>/	0.0	- 2.0<br>2.2	/	/<br>/	0.0	- 0.5<br>1.0	/	/<br>/
**3**	10.0$^{*, \#}$	7.0<br>10.0	5.0$^{*, \#}$	3.8<br>8.0	- 8.0*	- 9.0<br>- 3.0	- 9.0$^{*, \#}$	- 10.0<br>- 5.5	0.0	0.0<br>0.0	0.5	- 1.5<br>2.0	0.0	0.0<br>0.0	1.5	- 0.5<br>3.8
**5**	9.0$^{*, \#}$	9.0<br>10.0	6.0$^{*, \#}$	0.5<br>7.8	- 10.0*	- 10.0<br>- 3.0	- 10.0$^{*, \#}$	- 10.0<br>- 10.0	0.0	0.0<br>0.0	0.5	2.5<br>2.0	0.0	0.0<br>0.0	- 1.5	- 2.5<br>1.5
**7**	9.0*	7.0<br>10.0	6.0$^{*, \#}$	2.5<br>7.5	- 9.0$^{*, \#}$	- 10.0<br>- 5.0	- 10.0$^{*, \#}$	- 10.0<br>- 9.5	0.0	0.0<br>0.0	0.0	- 1.5<br>1.2	1.0	1.0<br>1.0	0.0	- 1.8<br>1.8
**14**	4.5	1.8<br>8.0	4.0	- 2.0<br>9.0	- 8.5$^{*, \#}$	- 9.8<br>- 5.8	- 10.0$^{*, \#}$	- 10.0<br>- 9.0	0.0	0.0<br>0.0	1.0	- 0.2<br>2.2	0.0	0.0<br>0.0	0.0	- 2.2<br>2.0
**21**	4.0	- 3.0<br>6.5	- 4.0	- 7.0<br>0.0	- 8.0$^{*, \#}$	- 8.5<br>- 6.0	- 10.0$^{*, \#}$	- 10.0<br>- 10.0	0.0	- 2.0<br>2.5	0.0	- 3.5<br>1.5	- 1.0	- 2.0<br>2.5	0.0	- 2.0<br>3.0

Data are presented as median ± (25th, 75th) percentile.

* Symbol indicating a significant (p<0.05) difference between 6-OHDA and SHAM groups.

$^{\#}$ Symbol indicating a significant (p<0.05) difference between time points with respect to pre-surgery condition (day 0).

Positive values indicate ipsilateral swings; negative values indicate contralateral swings.

Table S2. Summary of the turns/min assessed by the Rotational Test (RT) before (day 0) and after (days 1 to 21) the lesion for the 6-OHDA and SHAM groups, in drug-free (FREE) and after apomorphine (APO) administration for the single (S) and repeated (R) apomorphine administration protocols.

**Days**	**6-OHDA**								**SHAM**							
	**FREE**				**APO**				**FREE**				**APO**			
	**S**	**25th<br>75th**	**R**	**25th<br>75th**	**S**	**25th<br>75th**	**R**	**25th<br>75th**	**S**	**25th<br>75th**	**R**	**25th<br>75th**	**S**	**25th<br>75th**	**R**	**25th<br>75th**
**0**	0.0	0.0<br>0.0	/	/<br>/	0.0	0.0<br>0.0	/	/<br>/	0.0	0.0<br>0.0	/	/<br>/	0.0	0.0<br>0.0	/	/<br>/
**1**	$0.2^{*,\#}$	- 0.4<br>0.0	/	/<br>/	$- 3.6^{*,\#}$	- 4.6<br>- 2.4	/	/<br>/	0.0	0.0<br>0.1	/	/<br>/	0.0	0.0<br>0.0	/	/<br>/
**3**	0.3	- 0.6<br>0.0	$0.2^{\#}$	- 0.7<br>0.0	- 3.8	- 3.9<br>- 2.8	$- 7.4^{*,\#}$	- 9.4<br>- 5.1	- 0.2	- 0.2<br>- 0.2	0.0	0.0<br>0.1	0.0	0.0<br>0.0	0.0	0.0<br>0.1
**5**	0.3	- 0.9<br>0.0	$0.2^{\#}$	- 0.4<br>0.0	$- 8.2^{\#}$	- 9.6<br>- 0.3	$- 11.2^{*,\#}$	- 13.8<br>- 9.4	- 0.1	- 0.1<br>- 0.1	0.0	0.0<br>0.0	0.0	0.0<br>0.0	0.0	0.1<br>0.0
**7**	0.1	- 0.4<br>0.0	0.1	- 0.4<br>0.0	$- 10.8^{\#}$	- 10.9<br>- 5.4	$- 14.4^{*,\#}$	- 16.0<br>- 7.5	0.0	0.0<br>0.0	- 0.1	- 0.1<br>0.1	0.1	0.1<br>0.1	0.0	- 0.2<br>0.1
**14**	$0.3^{\#}$	- 0.5<br>- 0.3	$0.6^{\#}$	- 0.8<br>0.1	$- 8.9^{\#}$	- 12.3<br>- 7.9	$- 12.5^{*,\#}$	- 13.6<br>- 9.9	0.1	0.1<br>0.1	0.0	0.0<br>0.1	0.1	0.1<br>0.1	0.0	0.0<br>0.2
**21**	0.1	- 0.6<br>0.0	0.2	- 0.6<br>- 0.1	$- 8.8^{\#}$	- 10.8<br>- 1.6	$- 11.2^{*,\#}$	- 12.2<br>- 7.4	0.0	0.0<br>0.0	0.2	0.0<br>0.4	0.0	0.0<br>0.0	0.0	- 0.1<br>0.3

Data are presented as median ± (25th, 75th) percentile.

* Symbol indicating a significant (p<0.05) difference between 6-OHDA and SHAM groups.

\# Symbol indicating a significant (p<0.05) difference between time points with respect to pre-surgery condition (day 0).

Positive values indicate ipsilateral turns/min; negative values indicate contralateral turns/min.
